# The Immunomodulatory Effect and Clinical Efficacy of Daratumumab in a Patient With Cold Agglutinin Disease

**DOI:** 10.3389/fimmu.2021.649441

**Published:** 2021-03-01

**Authors:** Anna Zaninoni, Juri A. Giannotta, Anna Gallì, Rosangela Artuso, Paola Bianchi, Luca Malcovati, Wilma Barcellini, Bruno Fattizzo

**Affiliations:** ^1^Fondazione IRCCS Ca' Granda Ospedale Maggiore Policlinico, University of Milan, Milan, Italy; ^2^Department of Hematology Oncology, Fondazione IRCCS Policlinico San Matteo, Pavia, Italy; ^3^Medical Genetics Unit, Meyer Children's University Hospital, Florence, Italy; ^4^Department of Molecular Medicine, University of Pavia, Pavia, Italy

**Keywords:** cold agglutinin disease, daratumumab, cytokines, autoimmune diseases, anti-CD38 MoAb

## Abstract

Daratumumab is a monoclonal antibody directed against the transmembrane glycoprotein CD38 expressed on plasma cells and lymphoplasmocytes, with a proven efficacy in multiple myeloma. Here we show its clinical efficacy in a patient with cold agglutinin disease (CAD) relapsed after multiple lines of therapy. CAD is caused by cold reactive autoantibodies that induce complement mediated hemolysis and peripheral circulatory symptoms. The disease is also characterized by the presence of monoclonal IgM gammopathy and of a lymphoid bone marrow infiltration that benefits from B-cell targeting therapies (i.e., rituximab) but also from plasma cell directed therapies, such as proteasome inhibitors. In the patient described, we also show that daratumumab therapy influenced the dynamics of several immunoregulatory cytokine levels (IL-6, IL-10, IL-17, IFN-γ, TNF-α, TGF-β) indicating an immunomodulatory effect of the drug beyond plasma cell depletion. In addition, we provide a literature review on the use of daratumumab in autoimmune conditions, including multi-treated and refractory patients with autoimmune hemolytic anemia (both CAD and warm forms), Evans syndrome (association of autoimmune hemolytic anemia and immune thrombocytopenia) and non-hematologic autoimmune diseases, such as systemic lupus erythematosus and rheumatoid arthritis.

## Introduction

Cold agglutinin disease (CAD) is a rare form of autoimmune hemolytic anemia (AIHA) (1, 2) generally caused by an IgM autoantibody. The latter has an optimum temperature of reaction at 3–4°C and is able to fix complement with positivity of the direct antiglobulin test (DAT) for the C3d fraction. This leads to anemia of various degrees and to cold-induced circulatory symptoms that are present in the majority of cases irrespective of Hb levels. In more than 90% of patients a monoclonal IgMκ can be found, although other isotypes may be present. In addition, a lymphoid bone marrow infiltration, different from other non-Hodgkin lymphomas, has been demonstrated in the majority of cases. Molecular studies showed that *MYD88* mutation is always absent and that *KMT2D* and *CARD11* ones are present in a proportion of cases (3). Most therapies have been imported from lymphoproliferative syndromes, including the anti-CD20 monoclonal antibody (MoAb) rituximab, which is now the preferred first line treatment, since steroids are effective only at high unacceptable doses (4). Plasma cells directed therapies like bortezomib, a proteasome inhibitor used in multiple myeloma (MM), represent a novel approach in CAD patients with 30% response rate in a phase 2 clinical trial (5). Another interesting anti-MM treatment is daratumumab a human IgG1κ MoAb directed against the transmembrane glycoprotein CD38 (6, 7). Several reports highlighted the efficacy of daratumumab in different settings, including refractory and post-transplant warm AIHA (8–12), CAD (13), and Evans' syndrome (i.e., association of AIHA and immune thrombocytopenia) (14, 15). Besides these proofs of efficacy, the regulatory effect of daratumumab on immune response mediators such as cytokines is largely unknown. Here we describe the clinical efficacy of daratumumab in a patient with CAD relapsed after multiple lines of therapy, focusing on the dynamics of immunoregulatory cytokine levels. In addition, a literature review of daratumumab use in AIHA and other autoimmune diseases is provided.

## Patient and Methods

The patient has been regularly followed at our Center from the initial diagnosis (June 2011) until the time of writing. Clinical and laboratory data regarding CAD have been collected before each administration of daratumumab. The drug has been administered according to the currently approved schedule for MM: 16 mg/kg intravenously, weekly from 1st to 8th week, every 2 weeks from 9th to 24th week, and then every 4 weeks until disease progression. The patient signed informed consent and the study was conducted in accordance with the Helsinki declaration.

### Molecular Studies

DNA targeted next generation sequencing (NGS) was performed as previously described (16) using Illumina TruSight Myeloid Sequencing Panel (Illumina, San Diego, CA, USA) including 54 genes frequently mutated in myeloid disorders.

Functionally annotated variants were filtered according to the information retrieved from population databases (dbSNP, 1000 genome, ESP6500 and EXAC). The remaining variants were tagged as oncogenic based on the information derived from literature, Catalog of Somatic Mutations in Cancer (COSMIC) and on *in silico* prediction effect, as previously described (17). In addition, variants with a germline allele frequency were annotated using ClinVar and Online Mendelian Inheritance in Man and classified according to American College of Medical Genetics and Genomics and the Association for Molecular Pathology (ACMG/AMP) standards and guidelines for the interpretation of germline sequence variants (18).

Whole exome sequencing (WES) was performed using DNA extracted from peripheral blood by standard methods. The genomic DNA was sequenced with the platform NextSeq500 (Illumina Inc., San Diego, CA); reads were aligned with the human reference hg19 genome using Burrows–WheelerAligner (19), mapped and analyzed with the Integrative Genome Viewer software (2013 Broad Institute) (20). Downstream alignment processing (i.e., alignment, sorting, indexing, deduplication, and base quality score recalibration) was performed with the Genome Analysis Tool kit Unified Genotyper Module (GATK) (21), SAM tools (22), and Picard Tools (http://picard.sourceforge.net/). The GATK Unified Genotyper was used to obtain a set of single nucleotide variants and indel calls for the *KMT2D* and *CARD11* genes. All identified variants by WES have been classified as potentially pathogenic variants, VUS, or benign variants in agreement with the interpretation guidelines of the ACMG (18).

### Cytokine Studies

Biological samples for cytokine analysis have been collected prospectively since June 2019. The following cytokines have been tested in serum using high sensitivity elisa kits: interleukine (IL)-6, IL-10, IL-17, interferon (IFN)-γ, tumor necrosis factor (TNF)-α (High Sensitivity Elisa kits, eBioscience, Inc., Vienna Austria), and transforming growth factor (TGF)-β (Immunological Sciences, Rome, Italy). Patient's cytokine levels have been compared with median values of 40 healthy controls.

### Literature Review

A review of literature about daratumumab use in AIHA and other autoimmune diseases was performed by searching for indexed articles and published abstracts until December 2020 in MEDLINE via PubMed and the National Library of Medicine.

## Results

### Case Description

**Figure 1:**
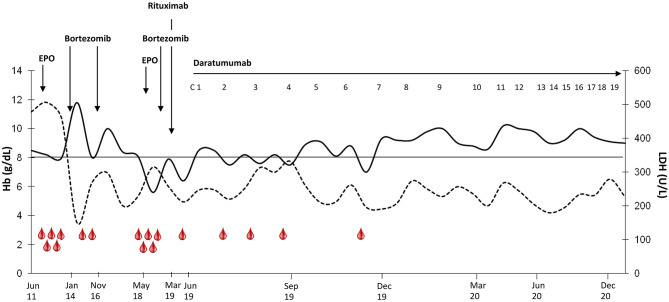
Hemoglobin and LDH values before and during treatment with daratumumab. Hb is shown as continuous line and LDH as dashed line. Transfusions are shown as red drops. Daratumumab cycles: 1–2, weekly administration; 3–8 fortnightly administration; 9–13, monthly administration. The drug has been administered according to the currently approved schedule for multiple myeloma: 16 mg/kg intravenously, weekly from 1st to 8th week, every 2 weeks from 9th to 24th week, and then every 4 weeks until disease progression.

A 59-year-old man presented in June 2011 with severe cold-related circulatory symptoms. Workup showed DAT-negative hemolytic anemia (Hb 8.5 g/dL, LDH 2xULN), splenomegaly (18 cm by ultrasound), and monoclonal IgG/k of 0.2 g/dL. Congenital causes were excluded, and more sensitive tests showed the presence of low-titer cold agglutinin (1:8), with DAT positivity after mitogen-stimulation (23). Bone marrow evaluation showed hypercellularity, with erythroid hyperplasia, dyserythropoiesis, and plasma cells infiltration of 5% (restricted for kappa light chain). Overall, the above-mentioned investigations led a diagnosis of CAD, although it usually requires a cold agglutinin titer >1:64 (2). As shown in Figure 1, the patient needed intense transfusion support, on average 2 red cell units every 15–20 days, despite the introduction of recombinant human erythropoietin (rhEPO, 40,000 U/week). In January 2014 the patient was enrolled in the CAD0111 study with bortezomib 1.3 mg/m2 IV on days 1, 4, 8, 11 (5). He reached a complete response lasting more than 2 years. A new bortezomib course was repeated in November 2016 with partial response lasting about 1 year. In May 2018, Hb levels dropped to 8.5 g/dL, again with disabling circulatory symptoms. Bone marrow re-evaluation was unchanged; a new course of bortezomib, as well as rhEPO, was ineffective. Thereafter, the patient returned transfusion-dependent, and rituximab was administered without response. A further bone marrow evaluation showed no erythroid hyperplasia, presence of dyserythropoiesis, reticulinic fibrosis, and a plasma cell infiltration of about 7% (restricted for kappa chain and CD38-expressing). FISH panel for MM and PCR analysis for *MYD88* mutation resulted negative. Moreover, whole exome sequencing analysis for *CARD11* and *KMT2D* did not found any pathogenic variants (3). To have a deep insight into mutational landscape of the CAD patient, we performed next generation sequencing at high depth of coverage (mean 4822x) and did not identify any pathogenic variant in the 54 tested genes. We only found a *GATA2* p.A286S variant, with a 50% allele frequency suggestive for germline origin that was considered of uncertain significance given a clinic history negative for GATA2-mutated syndrome and absence of criteria (ACMG/AMP standards and guidelines for the interpretation of germline sequence variants) (16, 18).

In June 2019 the patient was still severely anemic (Hb 7 g/dL), with transfusion dependence (2–3 RBC units per month) and hemolytic markers alteration (LDH 1.3 × ULN, unconjugated bilirubin 3.1 mg/dL, reticulocytes 144 × 10^9^/L, and haptoglobin consumption). Daratumumab was started with progressive improvement of anemia, transfusion avoidance, and disappearance of circulatory symptoms (Figure 1). Therapy was well-tolerated, and the patient reported no side effects, particularly no infusion reactions or infections, notwithstanding the decrease of IgA, IgG and IgM serum levels during anti-CD38 treatment (94 mg/dL pre-treatment → 16 mg/dL at month +16, 1,083 → 808 mg/dL, and 73 → 18 mg/dL, respectively). BM evaluation after 6 cycles showed normal cellularity, with 2% plasma cells infiltration (phenotypically unchanged compared to pre-daratumumab evaluation). The low titer of cold agglutinins and monoclonal IgG/k levels were unchanged. At the time of writing, the patient is still receiving daratumumab with a stable clinical picture (Hb 10.2 g/dL, LDH 0.8 × ULN, unconjugated bilirubin 0.8 mg/dL, normal haptoglobin).

### Cytokine Studies

**Figure 2:**
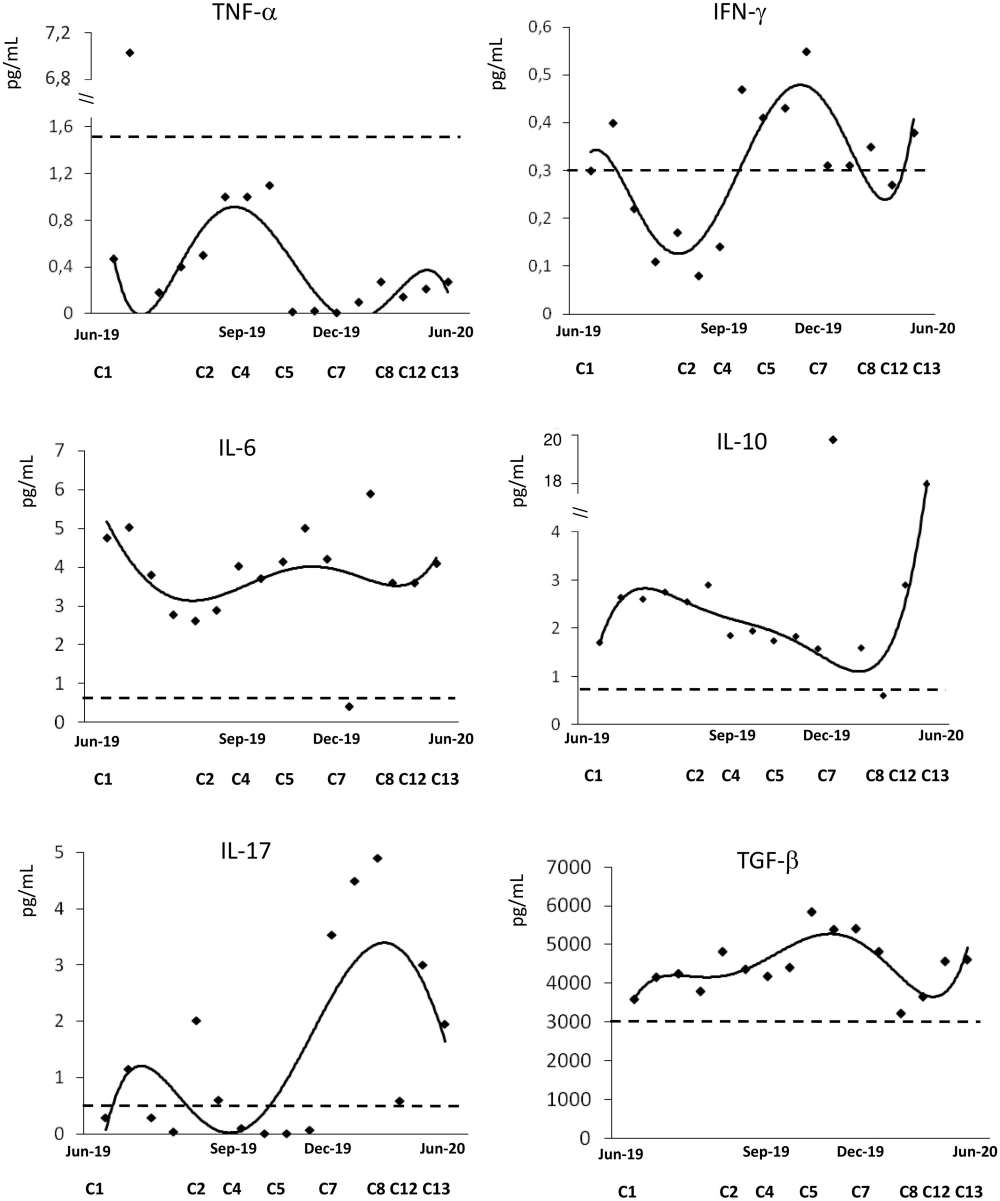
Cytokine profile during treatment with daratumumab. Interleukin (IL)-6, IL-10, IL-17, interferon (IFN)-γ, tumor necrosis factor (TNF)-α, transforming growth factor (TGF)-β. Dashed line indicates the median values of 40 healthy controls.

Figure 2 shows the dynamics of cytokine levels before and during daratumumab treatment. The T helper1 (Th1) cytokine TNF-α was lower than the median of controls, and negatively correlated with Hb (*r* = −0.57, *p* = 0.04). IFN-γ levels showed a bimodal pattern over time, with an initial decrease followed by a rise and a subsequent normalization; its levels negatively correlated with LDH (*r* = −0.56, *p* = 0.046). More interestingly, the T helper 2 (Th2) cytokines IL-6 and IL-10 levels were higher than in controls, with a remarkable increase of the latter after cycle 7. Similarly, IL-17 was initially around normal values and markedly increased by cycle 7, when clinical response consolidated. Consistently, IL-17 positively correlated with Hb (*r* = 0.66, *p* = 0.01). Finally, the inhibitory cytokine TGF-β was constantly over the normal median of controls.

### Review of the Literature

Table 1 summarizes available evidence about daratumumab use in 12 AIHAs. The majority of articles are case reports of warm AIHA post-hematopoietic stem cell transplant (HSCT) or associated with immune thrombocytopenia (ITP, *N* = 2), and almost all patients were children. Of note, daratumumab was administered as third or further therapy lines, and in 4 cases after a mean of 7.5 treatments. Daratumumab dose was the same as in MM, whilst the number of administrations varied greatly from 1 to 11, mean 4.9 doses. Responses were observed in all but 1 case and consisted of Hb amelioration and reduction of transfusion; 2 responses were only transient, and 1 of them had a fatal outcome. Of note, time to response was generally very short, the majority of responses being observed within 2 weeks. The only CAD patient reported required many daratumumab cycles to achieve a response, both hematological and on disabling circulatory symptoms (13). Table 2 reports data of daratumumab use in other autoimmune conditions and includes preclinical and clinical evidence. The former encompasses a large study on rheumatoid arthritis and systemic lupus erythematosus (SLE), providing evidence of CD38 expression on plasma cell/plasma blasts from these patients (24). In a further study, daratumumab treatment was shown to reduce autoantibodies (antinuclear, anti-neutrophil cytoplasmic antibodies, and rheumatoid factor) in patients with MM (25). Finally, anti-CD38 treatment ameliorated autoimmune disease in an animal model of cynomolgus monkeys (32). As regards clinical reports, few and heterogeneous conditions are described, including two post-HSCT cytopenias (pure red cell aplasia and ITP) (27, 28), 2 SLE (29), and 2 autoimmune encephalitis (30, 31). All patients were adult and heavily pre-treated. As observed for AIHA, most patients responded in a short time, with a variable number of cycles. Notably, in the two patients with SLE, daratumumab promoted depletion of autoreactive long-lived plasma cells and was associated with a reduction in interferon type I activity and with modulation of effector T-cell responses (29).

**Table 1:** Review of the literature about daratumumab use in warm autoimmune hemolytic anemia (wAIHA), cold agglutinin disease (CAD), and Evans' syndrome.

**Disease**	**N of patients**	**Previous treatments**	**Dara schedule**	**Time to response**	**Main finding**	**References**
wAIHA post HSCT for ALL	1 (19-year-old woman)	Steroids, rituximab 2 courses, high-dose cyclophosphamide, rabbit anti-thymocyte globulin, alemtuzumab, bortezomib 2 courses, mycophenolate mofetil, sirolimus, and ibrutinib	16 mg/kg single infusion	2–4 weeks	Daratumumab dramatically decreased red cells transfusion in a refractory patient	(8)
wAIHA post HSCT for ALL	3 (pediatric cases)	Steroids, rituximab, PEX, bortezomib, MMF, sirolimus, ATG, CyA, ibrutinib, eculizumab	16 mg/kg Pt1: 11 doses in 49 days Pt2: 4 in 17 days Pt3: 7 doses in 43 days	Not reported	Daratumumab was effective in two out of three pediatric patients with heavily pre-treated patients, 1 died after a transient response	(9)
wAIHA during HSCT for Langerhans cell histiocytosis	1 (pediatric case)	Steroids, rituximab, IVIG, Whole blood exchange	16 mg/kg/day ×2 doses	3 days	Daratumumab contributed to anemia recovery in a refractory patient	(10)
wAIHA	1 (60-year-old woman)	Steroids, rituximab	16 mg/kg/week ×4 doses	10 weeks	Daratumumab induced a 20 weeks lasting response in a pre-treated patient	(12)
wAIHA post HSCT	3 out of 20 pediatric AIC cases (case-control study)	Corticosteroids (75%) and rituximab (55%)	Not reported	Mean 1,069 days	Daratumumab was started at days +245, +1,028, and +2,016 following HSCT. 2 patients responded and had resolution of B-cell aplasia 759 and 1,380 days following daratumumab	(11)
Evans syndrome post HSCT for AA (wAIHA+ITP)	1 (35-year-old woman)	Steroids, IVIG, CyA, eltrombopag, rituximab, bortezomib,	16 mg/kg/week ×4 doses	2 weeks	Daratumumab induced rapid recovery of highly pre-treated adult patient	(15)
Evans syndrome post HSCT for MF (wAIHA+ITP)	1 (pediatric case)	Steroids, IVIG, rtixuimab, abatacept, bortezomib	16 mg/kg/week ×6 doses	Immediate	Daratumumab induced immediate response in a heavily pre-treated child	(14)
CAD secondary to LPL	1 (48-year-old man)	Steroids, rituximab, Bortezomib-cyclophosphamide-prednisolone	16 mg/kg/week for week 1–8, 16 mg/kg fortnight for week 9–24, then 16 mg/kg/month	2 weeks	Daratumumab resolved cold agglutinin peripheral symptoms and increased Hb values; cold agglutinin titer were also reduced	(13)

*wAIHA, warm autoimmune hemolytic anemia; HSCT, hematopoietic stem cell transplant; ALL, acute lymphoblastic leukemia; AA, aplastic anemia; MF, myelofibrosis; ITP, immune thrombocytopenia; LPL, lymphoplasmocytic lymphoma*.

**Table 2:** Literature review of daratumumab use in autoimmune conditions other than autoimmune hemolytic anemia.

**Disease**	**N of patients**	**Previous treatments**	**Dara schedule**	**Time to response**	**Main finding**	**References**
**Preclinical evidence**						
Rheumatoid arthritis and systemic lupus erythematosus	152 human samples, *ex vivo* study	-	Not applicable	-	Plasma cell/plasmablast-related genes CD38, XBP1, IRF4, PRDM1, IGJ, and TNFSF13B are significantly up-regulated in patients with rheumatoid arthritis and systemic lupus erythematosus	(24)
MM patients with autoantibodies	6 human samples, *ex vivo* study	MM treatments	16 mg/kg/week ×4 doses	After 1st infusion	Autoantibodies rapidly disappeared in 5 out of 6 patients during daratumumab treatment	(25)
Collagen induced arthritis	Animal model, *in vivo* study	–	Not applicable	–	Targeting CD38-expressing leukocytes with a cytolytic antibody can ameliorate autoimmune disease in cynomolgus monkeys	(26)
**Clinical evidence**						
PRCA post HSCT	1 (72-year-old man)	Steroids, rituximab, recombinant erythropoietin	16 mg/kg/week ×6 doses	1 week	Daratumumab induced recovery of anemia, abolishing transfusion requirement	(27)
ITP post HSCT for MDS	1 (60-year-old man)	corticosteroids, vincristine, IVIG, rituximab, PEX, splenectomy, romiplostim, eltrombopag, danazol	16 mg/kg/week ×4 doses	8 weeks	Daratumumab induced sustained complete remission in a multi-refractory patient	(28)
Systemic lupus erythematosus	2 (50- and 32-year old women)	Steroids, MMF, CyA, CTX, bortezomib, belimumab, rituximab, azathioprine, MTX, hydroxychloroquine, PEX, IVIG	16 mg/kg/week ×4 doses	Gradual amelioration in 12 months and 10 weeks, respectively	Daratumumab provided clinical and serologic responses in two refractory patients. Daratumumab promoted depletion of autoreactive long-lived plasma cells and was associated with a reduction in interferon type I activity and with modulation of effector T-cell responses	(29)
anti-CASPR2 encephalitis	1 (60-year-old man)	Steroids, PEX, immunoadsorption, IVIG, rituximab, bortezomib	16 mg/kg/week ×13 doses	3 months	Daratumumab ameliorated clinical and laboratory findings of autoimmune encephalitis	(30)
Anti-NMDA encephalitis	1 (20-year-old woman)	steroids, PEX, IVIG, rituximab, bortezomib	16 mg/kg/week ×8 doses, then fortnightly ×2 doses	2 months	Daratumumab ameliorated clinical status and the patient was able to resume everyday and self-care activities	(31)

*MM, multiple myeloma; PRCA, pure red cell aplasia; HSCT, hematopoietic stem cell transplant; ITP, immune thrombocytopenia; MDS, myelodsyplastic syndrome; CASPR2, Contactin-associated protein-like 2, NMDA, N-methyl-D-aspartate*.

## Discussion

Here we report the efficacy of daratumumab therapy in a patient with CAD and show for the first time its immunomodulatory effect on several cytokines. Even though the patient did not experience a complete response as per published criteria (4), Hb levels increased of 3 g/dL allowing transfusion independence and disabling circulatory symptoms disappeared. The case presented had a long history of multi treated CAD with severe transfusion-dependent anemia, low cold agglutinin titer, and IgG monoclonal gammopathy. Molecular studies did not further inform the diagnosis. As in our patient, the literature review highlighted the efficacy of daratumumab in several clinical settings, including multi-refractory CAD and wAIHA/Evans syndrome, mainly occurring after hematopoietic stem cell transplant (8–15). Today, no effective second line treatment has been licensed for CAD and novel drugs targeting the complement cascade or the antibody producing B-cell clone are under active investigation. Regarding B-cell directed therapies, small molecules targeting the B-cell receptor showed promising results in AIHA secondary to chronic lymphocytic leukemia and are under active study in primary cases (33). Concerning anti-plasma cells therapies, it has been postulated that bortezomib and daratumumab target both short- and long-lived plasma cells that produce the autoantibodies. Since CD38 is expressed on both plasma cells and lymphoplasmocytes, daratumumab may be a double-targeting strategy for refractory/relapsing CAD, contrarily to rituximab, which does not affect long-lived plasma cells and was ineffective in our patient. Furthermore, our patient progressively lost response to bortezomib, which, although active on long-lived plasma cells, does not target CD38-positive cells. We may hypothesize that previous bortezomib therapy selected a resistant plasma cell-clone, which eventually responded to anti-CD38 treatment.

The effect of daratumumab in our patient seems to be different than that observed in multiple myeloma. As a matter of fact, the patient did not show a plasma cell burden >10% within the BM at daratumumab initiation. Additionally, the response of hemolysis and hemoglobin were far slower than those observed in MM (median 1 month, 0.9–5.6). This suggests that daratumumab may exert an immunomodulatory effect, either because of or in addition to plasma cell depletion, similarly to what observed for rituximab in AIHA.

Regarding cytokine studies, patient's IL-6 and IL-10 levels were increased compared to controls, consistently with the antibody-mediated mechanism of CAD and with the several reports of increased levels in MM (33, 34). Additionally, IL-10 is a negative regulator of Th1 cytokines, and might have accounted for the reduced levels of TNF-α and IFN-γ observed in our patient in the first phase of daratumumab treatment. Contrarily, the normalization/increase in IFN-γ levels found after several cycles might be attributed to the engagement of this cytokine in daratumumab-mediated cytotoxicity against plasma cells. Additionally, daratumumab may exert its anti-plasma cells effect through the engagement of natural killer (NK) cells, as recently reported in MM (35).

Concerning regulatory cytokines, TGF-β is a multifunctional cytokine that plays a crucial role in stem cell differentiation, T-cell differentiation, and inflammatory processes. Moreover, TGF-β favors the differentiation of Th17 subset. This amplifies the pro-inflammatory and autoimmune response by overproduction of IL-17 and may also boost cytotoxic activity. In our patient TGF-β was higher than controls at all time points tested, likely contributing to IL-17 overproduction. This was particularly evident after several cycles of daratumumab, possibly potentiating daratumumab-induced cytotoxicity against plasma cells.

In conclusion, our case, along with previous reports, indicates that daratumumab is effective in CAD in ameliorating anemia and improving disabling circulatory symptoms, providing an additional therapeutic option for the refractory disease. Furthermore, we speculate about the possible immunomodulatory activity of daratumumab in addition to plasma cell depletion.

## Data Availability Statement

The raw data supporting the conclusions of this article will be made available by the authors, without undue reservation.

## Ethics Statement

The studies involving human participants were reviewed and approved by Comitato Etico Milano Area 2. The patients/participants provided their written informed consent to participate in this study.

## Author Contributions

JG, WB, and BF followed the patient and wrote the manuscript. AZ performed cytokine analysis and wrote the manuscript. AG and LM performed next generation sequencing tests and revised the manuscript for important intellectual content. RA performed *KMT2D* and *CARD11* sequencing. PB revised the manuscript for important intellectual content. All authors contributed to the article and approved the submitted version.

## Conflict of Interest

The authors declare that the research was conducted in the absence of any commercial or financial relationships that could be construed as a potential conflict of interest.
